# Supplementary figures and images for: Seasonal patterns of bird and bat collision fatalities at wind turbines
Source: PLoS One. 2023 May 10;18(5):e0284778. doi: 10.1371/journal.pone.0284778 (PMC10171668; doi:10.1371/journal.pone.0284778)

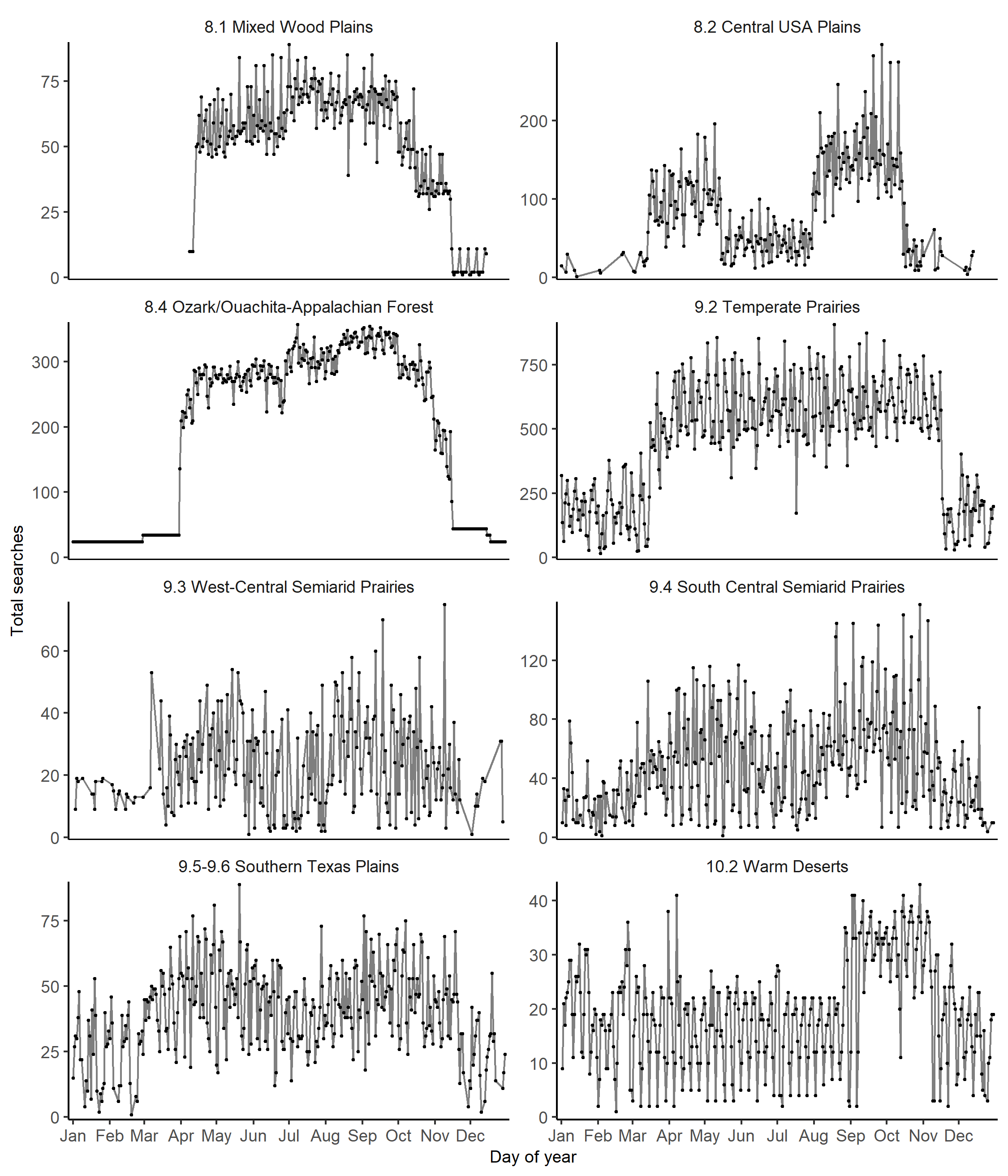

Supplement: S1 Fig — (TIF) [file pone.0284778.s001.tif]

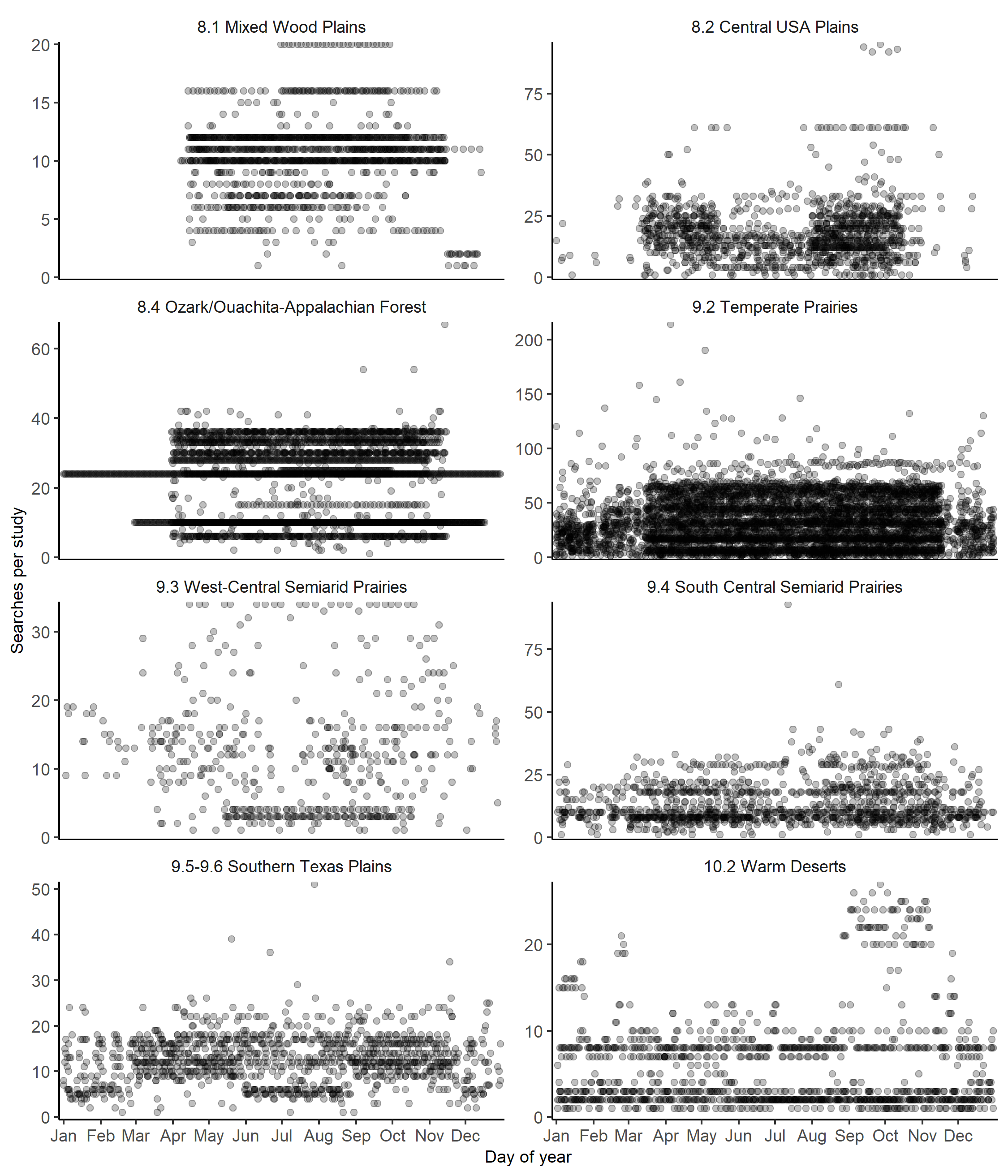

Supplement: S2 Fig — (TIF) [file pone.0284778.s002.tif]
