# Supplementary material for: Seasonal patterns of bird and bat collision fatalities at wind turbines
Source: PLoS One. 2023 May 10;18(5):e0284778. doi: 10.1371/journal.pone.0284778 (PMC10171668; doi:10.1371/journal.pone.0284778)
Supplement: S1 Table — (DOCX) [file pone.0284778.s003.docx]

**S1 Table.** **Bird species included in the Grassland, Soaring, and Woodland guilds**.

| Model group | Species | Scientific name |
| --- | --- | --- |
| Grassland | American Pipit | *Anthus rubescens* |
| Grassland | Bobolink | *Dolichonyx oryzivorus* |
| Grassland | Dickcissel | *Spiza americana* |
| Grassland | Eastern Kingbird | *Tyrannus tyrannus* |
| Grassland | Eastern Meadowlark | *Sturnella magna* |
| Grassland | Field Sparrow | *Spizella pusilla* |
| Grassland | Grasshopper Sparrow | *Ammodramus savannarum* |
| Grassland | Horned Lark | *Eremophila alpestris* |
| Grassland | Lapland Longspur | *Calcarius lapponicus* |
| Grassland | Lark Sparrow | *Chondestes grammacus* |
| Grassland | LeConte’s Sparrow | *Ammodramus leconteii* |
| Grassland | Thick-billed Longspur | *Rhynchophanes mccownii* |
| Grassland | Savannah Sparrow | *Passerculus sandwichensis* |
| Grassland | Sedge Wren | *Cistothorus platensis* |
| Grassland | Vesper Sparrow | *Pooecetes gramineus* |
| Grassland | Western Kingbird | *Tyrannus verticalis* |
| Grassland | Western Meadowlark | *Sturnella neglecta* |
| Soaring | American Kestrel | *Falco sparverius* |
| Soaring | Black Vulture | *Coragyps atratus* |
| Soaring | Common Raven | *Corvus corax* |
| Soaring | Cooper’s Hawk | *Accipiter cooperii* |
| Soaring | Crested Caracara | *Caracara cheriway* |
| Soaring | Ferruginous Hawk | *Buteo regalis* |
| Soaring | Golden Eagle | *Aquila chrysaetos* |
| Soaring | Harris’s Hawk | *Parabuteo unicinctus* |
| Soaring | Northern Harrier | *Circus cyaneus* |
| Soaring | Peregrine Falcon | *Falco peregrinus* |
| Soaring | Prairie Falcon | *Falco mexicanus* |
| Soaring | Red-tailed Hawk | *Buteo jamaicensis* |
| Soaring | Sharp-shinned Hawk | *Accipiter striatus* |
| Soaring | Swainson’s Hawk | *Buteo swainsoni* |
| Soaring | Turkey Vulture | *Cathartes aura* |
| Soaring | White-tailed Hawk | *Buteo albicaudatus* |
| Soaring | White-tailed Kite | *Elanus leucurus* |
| Woodland | Acadian Flycatcher | *Empidonax virescens* |
| Woodland | American Redstart | *Setophaga ruticilla* |
| Woodland | Baltimore Oriole | *Icterus galbula* |
| Woodland | Bay-breasted Warbler | *Setophaga castanea* |
| Woodland | Black-and-white Warbler | *Mniotilta varia* |
| Woodland | Black-billed Cuckoo | *Coccyzus erythropthalmus* |
| Woodland | Black-throated Blue Warbler | *Setophaga caerulescens* |
| Woodland | Black-throated Gray Warbler | *Setophaga nigrescens* |
| Woodland | Black-throated Green Warbler | *Setophaga virens* |
| Woodland | Blackburnian Warbler | *Setophaga fusca* |
| Woodland | Blackpoll Warbler | *Setophaga striata* |
| Woodland | Blue-headed Vireo | *Vireo solitarius* |
| Woodland | Cape May Warbler | *Setophaga tigrina* |
| Woodland | Cerulean Warbler | *Setophaga cerulea* |
| Woodland | Chuck-will’s-widow | *Antrostomus carolinensis* |
| Woodland | Connecticut Warbler | *Oporornis agilis* |
| Woodland | Golden-crowned Kinglet | *Regulus satrapa* |
| Woodland | Gray-cheeked Thrush | *Catharus minimus* |
| Woodland | Great Crested Flycatcher | *Myiarchus crinitus* |
| Woodland | Kentucky Warbler | *Geothlypis formosa* |
| Woodland | Least Flycatcher | *Empidonax minimus* |
| Woodland | Magnolia Warbler | *Setophaga magnolia* |
| Woodland | Nashville Warbler | *Oreothlypis ruficapilla* |
| Woodland | Northern Parula | *Setophaga americana* |
| Woodland | Northern Waterthrush | *Parkesia noveboracensis* |
| Woodland | Orchard Oriole | *Icterus spurius* |
| Woodland | Ovenbird | *Seiurus aurocapilla* |
| Woodland | Philadelphia Vireo | *Vireo philadelphicus* |
| Woodland | Pine Warbler | *Setophaga pinus* |
| Woodland | Red-eyed Vireo | *Vireo olivaceus* |
| Woodland | Rose-breasted Grosbeak | *Pheucticus ludovicianus* |
| Woodland | Ruby-crowned Kinglet | *Regulus calendula* |
| Woodland | Scarlet Tanager | *Piranga olivacea* |
| Woodland | Swainson’s Thrush | *Catharus ustulatus* |
| Woodland | Tennessee Warbler | *Oreothlypis peregrina* |
| Woodland | Townsend’s Warbler | *Setophaga townsendi* |
| Woodland | Veery | *Catharus fuscescens* |
| Woodland | Western Tanager | *Piranga ludoviciana* |
| Woodland | Western Wood-Pewee | *Contopus sordidulus* |
| Woodland | Willow Flycatcher | *Empidonax traillii* |
| Woodland | Wilson’s Warbler | *Cardellina pusilla* |
| Woodland | Wood Thrush | *Hylocichla mustelina* |
| Woodland | Yellow-billed Cuckoo | *Coccyzus americanus* |
| Woodland | Yellow-rumped Warbler | *Setophaga coronata* |
| Woodland | Yellow-throated Vireo | *Vireo flavifrons* |
