# Supplementary material for: Seasonal patterns of bird and bat collision fatalities at wind turbines
Source: PLoS One. 2023 May 10;18(5):e0284778. doi: 10.1371/journal.pone.0284778 (PMC10171668; doi:10.1371/journal.pone.0284778)
Supplement: S2 Table — (DOCX) [file pone.0284778.s004.docx]

#### S2 Table. Summary of data available for model development for all-bat and all-bird models.

| Region  code | Ecoregion | Species group | Fatalities | Search days | Searches | Studies |
| --- | --- | --- | --- | --- | --- | --- |
| 8.1 | Mixed Wood Plains | Bats | 466 | 1228 | 12854 | 8 |
| 8.1 | Mixed Wood Plains | Birds | 217 | 1228 | 12854 | 8 |
| 8.2 | Central USA Plains | Bats | 1414 | 1253 | 22539 | 18 |
| 8.2 | Central USA Plains | Birds | 251 | 1253 | 22539 | 18 |
| 8.4 | Ozark/Ouachita-Appalachian Forests | Bats | 2385 | 3204 | 69427 | 17 |
| 8.4 | Ozark/Ouachita-Appalachian Forests | Birds | 818 | 3204 | 69427 | 17 |
| 9.2 | Temperate Prairies | Bats | 3718 | 5040 | 164468 | 33 |
| 9.2 | Temperate Prairies | Birds | 1030 | 5040 | 164468 | 33 |
| 9.3 | West-Central Semiarid Prairies | Bats | 227 | 488 | 5801 | 9 |
| 9.3 | West-Central Semiarid Prairies | Birds | 126 | 488 | 5801 | 9 |
| 9.4 | South Central Semiarid Prairies | Bats | 381 | 1218 | 16531 | 18 |
| 9.4 | South Central Semiarid Prairies | Birds | 478 | 1218 | 16531 | 18 |
| 9.5-9.6 | Southern Texas Plains | Bats | 1673 | 1107 | 13892 | 6 |
| 9.5-9.6 | Southern Texas Plains | Birds | 590 | 1107 | 13892 | 6 |
| 10.2 | Warm Deserts | Bats | 27 | 1183 | 6998 | 5 |
| 10.2 | Warm Deserts | Birds | 279 | 1183 | 6998 | 5 |
