# Supplementary material for: Seasonal patterns of bird and bat collision fatalities at wind turbines
Source: PLoS One. 2023 May 10;18(5):e0284778. doi: 10.1371/journal.pone.0284778 (PMC10171668; doi:10.1371/journal.pone.0284778)
Supplement: S3 Table — (DOCX) [file pone.0284778.s005.docx]

#### S3 Table. Summary of data available for model development in bat species and bird guild models.

| Region code | Ecoregion | Species/guild | Fatalities | Search days | Searches | Studies |
| --- | --- | --- | --- | --- | --- | --- |
| 8.1 | Mixed Wood Plains | Big brown bat | 21 | 1228 | 12854 | 8 |
| 8.1 | Mixed Wood Plains | Eastern red bat | 108 | 1228 | 12854 | 8 |
| 8.1 | Mixed Wood Plains | Grassland | 11 | 1228 | 12854 | 8 |
| 8.1 | Mixed Wood Plains | Hoary bat | 206 | 1228 | 12854 | 8 |
| 8.1 | Mixed Wood Plains | Mexican free-tailed bat | 0 | 1228 | 12854 | 8 |
| 8.1 | Mixed Wood Plains | Other bats | 30 | 1228 | 12854 | 8 |
| 8.1 | Mixed Wood Plains | Silver-haired bat | 96 | 1228 | 12854 | 8 |
| 8.1 | Mixed Wood Plains | Soaring | 6 | 1228 | 12854 | 8 |
| 8.1 | Mixed Wood Plains | Woodland | 84 | 1228 | 12854 | 8 |
| 8.2 | Central USA Plains | Big brown bat | 182 | 1253 | 22539 | 18 |
| 8.2 | Central USA Plains | Eastern red bat | 658 | 1253 | 22539 | 18 |
| 8.2 | Central USA Plains | Grassland | 54 | 1253 | 22539 | 18 |
| 8.2 | Central USA Plains | Hoary bat | 211 | 1253 | 22539 | 18 |
| 8.2 | Central USA Plains | Mexican free-tailed bat | 0 | 1253 | 22539 | 18 |
| 8.2 | Central USA Plains | Other bats | 20 | 1253 | 22539 | 18 |
| 8.2 | Central USA Plains | Silver-haired bat | 336 | 1253 | 22539 | 18 |
| 8.2 | Central USA Plains | Soaring | 20 | 1253 | 22539 | 18 |
| 8.2 | Central USA Plains | Woodland | 33 | 1253 | 22539 | 18 |
| 8.4 | Ozark/Ouachita-Appalachian Forests | Big brown bat | 108 | 3204 | 69427 | 17 |
| 8.4 | Ozark/Ouachita-Appalachian Forests | Eastern red bat | 928 | 3204 | 69427 | 17 |
| 8.4 | Ozark/Ouachita-Appalachian Forests | Grassland | 9 | 3204 | 69427 | 17 |
| 8.4 | Ozark/Ouachita-Appalachian Forests | Hoary bat | 832 | 3204 | 69427 | 17 |
| 8.4 | Ozark/Ouachita-Appalachian Forests | Mexican free-tailed bat | 0 | 3204 | 69427 | 17 |
| 8.4 | Ozark/Ouachita-Appalachian Forests | Other bats | 185 | 3204 | 69427 | 17 |
| 8.4 | Ozark/Ouachita-Appalachian Forests | Silver-haired bat | 318 | 3204 | 69427 | 17 |
| 8.4 | Ozark/Ouachita-Appalachian Forests | Soaring | 15 | 3204 | 69427 | 17 |
| 8.4 | Ozark/Ouachita-Appalachian Forests | Woodland | 522 | 3204 | 69427 | 17 |
| 9.2 | Temperate Prairies | Big brown bat | 519 | 5040 | 164468 | 33 |
| 9.2 | Temperate Prairies | Eastern red bat | 1466 | 5040 | 164468 | 33 |
| 9.2 | Temperate Prairies | Grassland | 106 | 5040 | 164468 | 33 |
| 9.2 | Temperate Prairies | Hoary bat | 1221 | 5040 | 164468 | 33 |
| 9.2 | Temperate Prairies | Mexican free-tailed bat | 0 | 5040 | 164468 | 33 |
| 9.2 | Temperate Prairies | Other bats | 207 | 5040 | 164468 | 33 |
| 9.2 | Temperate Prairies | Silver-haired bat | 293 | 5040 | 164468 | 33 |
| 9.2 | Temperate Prairies | Soaring | 72 | 5040 | 164468 | 33 |
| 9.2 | Temperate Prairies | Woodland | 233 | 5040 | 164468 | 33 |
| 9.3 | West-Central Semiarid Prairies | Big brown bat | 2 | 488 | 5801 | 9 |
| 9.3 | West-Central Semiarid Prairies | Eastern red bat | 13 | 488 | 5801 | 9 |
| 9.3 | West-Central Semiarid Prairies | Grassland | 37 | 488 | 5801 | 9 |
| 9.3 | West-Central Semiarid Prairies | Hoary bat | 119 | 488 | 5801 | 9 |
| 9.3 | West-Central Semiarid Prairies | Mexican free-tailed bat | 0 | 488 | 5801 | 9 |
| 9.3 | West-Central Semiarid Prairies | Other bats | 0 | 488 | 5801 | 9 |
| 9.3 | West-Central Semiarid Prairies | Silver-haired bat | 76 | 488 | 5801 | 9 |
| 9.3 | West-Central Semiarid Prairies | Soaring | 6 | 488 | 5801 | 9 |
| 9.3 | West-Central Semiarid Prairies | Woodland | 6 | 488 | 5801 | 9 |
| 9.4 | South Central Semiarid Prairies | Big brown bat | 8 | 1209 | 16471 | 17 |
| 9.4 | South Central Semiarid Prairies | Eastern red bat | 68 | 1209 | 16471 | 17 |
| 9.4 | South Central Semiarid Prairies | Grassland | 210 | 1218 | 16531 | 18 |
| 9.4 | South Central Semiarid Prairies | Hoary bat | 136 | 1209 | 16471 | 17 |
| 9.4 | South Central Semiarid Prairies | Mexican free-tailed bat | 104 | 1209 | 16471 | 17 |
| 9.4 | South Central Semiarid Prairies | Other bats | 40 | 1209 | 16471 | 17 |
| 9.4 | South Central Semiarid Prairies | Silver-haired bat | 5 | 1209 | 16471 | 17 |
| 9.4 | South Central Semiarid Prairies | Soaring | 44 | 1218 | 16531 | 18 |
| 9.4 | South Central Semiarid Prairies | Woodland | 14 | 1218 | 16531 | 18 |
| 9.5-9.6 | Southern Texas Plains | Big brown bat | 1 | 1107 | 13892 | 6 |
| 9.5-9.6 | Southern Texas Plains | Eastern red bat | 0 | 1107 | 13892 | 6 |
| 9.5-9.6 | Southern Texas Plains | Grassland | 74 | 1107 | 13892 | 6 |
| 9.5-9.6 | Southern Texas Plains | Hoary bat | 28 | 1107 | 13892 | 6 |
| 9.5-9.6 | Southern Texas Plains | Mexican free-tailed bat | 717 | 1107 | 13892 | 6 |
| 9.5-9.6 | Southern Texas Plains | Other bats | 666 | 1107 | 13892 | 6 |
| 9.5-9.6 | Southern Texas Plains | Silver-haired bat | 0 | 1107 | 13892 | 6 |
| 9.5-9.6 | Southern Texas Plains | Soaring | 42 | 1107 | 13892 | 6 |
| 9.5-9.6 | Southern Texas Plains | Woodland | 31 | 1107 | 13892 | 6 |
| 10.2 | Warm Deserts | Grassland | 44 | 1183 | 6998 | 5 |
| 10.2 | Warm Deserts | Soaring | 13 | 1183 | 6998 | 5 |
| 10.2 | Warm Deserts | Woodland | 30 | 1183 | 6998 | 5 |
