# Supplementary material for: Seasonal patterns of bird and bat collision fatalities at wind turbines
Source: PLoS One. 2023 May 10;18(5):e0284778. doi: 10.1371/journal.pone.0284778 (PMC10171668; doi:10.1371/journal.pone.0284778)
Supplement: S4 Table — (DOCX) [file pone.0284778.s006.docx]

#### S4 Table. Model selection results for base models (without grouping data by species).

| Model | AIC | ΔAIC |
| --- | --- | --- |
| carcasses ~ s(day) + re(site) + re(year) + offset(searches) | 32308 | 0 |
| carcasses ~ s(day) + re(site) + offset(searches) | 32434 | 126.4 |
| carcasses ~ s(day) + re(year) + offset(searches) | 35755 | 3447 |
| carcasses ~ s(day) + offset(searches) | 36201 | 3893 |
| s= smooth term; re=random effect |  |  |
