# Supplementary material for: Seasonal patterns of bird and bat collision fatalities at wind turbines
Source: PLoS One. 2023 May 10;18(5):e0284778. doi: 10.1371/journal.pone.0284778 (PMC10171668; doi:10.1371/journal.pone.0284778)
Supplement: S5 Table — (DOCX) [file pone.0284778.s007.docx]

#### S5 Table. Model selection results for all-bat/all-bird models.

| Model | AIC | ΔAIC |
| --- | --- | --- |
| carcasses ~ s(day, by = species group:ecoregion) + species group * ecoregion + re(site) + re(year) + offset(searches) | 40103 | 0 |
| carcasses ~ s(day, by = species group) + species group + s(day, by = ecoregion) + ecoregion + re(site) + re(year) + offset(searches) | 40851 | 747.8 |
| carcasses ~ s(day, by = species group) + species group + re(site) + re(year) + offset(searches) | 41476 | 1373 |
| carcasses ~ s(day, by = ecoregion) + ecoregion + re(site) + re(year) + offset(searches) | 43500 | 3397 |
| carcasses ~ s(day) + re(site) + re(year) + offset(searches) | 44081 | 3978 |
| carcasses ~ s(day) + re(site) + offset(searches) | 44191 | 4088 |
| carcasses ~ s(day) + re(year) + offset(searches) | 47447 | 7344 |
| carcasses ~ s(day) + offset(searches) | 47913 | 7810 |
| s=smooth term; re=random effect |  |  |
