# Supplementary material for: Seasonal patterns of bird and bat collision fatalities at wind turbines
Source: PLoS One. 2023 May 10;18(5):e0284778. doi: 10.1371/journal.pone.0284778 (PMC10171668; doi:10.1371/journal.pone.0284778)
Supplement: S6 Table — (DOCX) [file pone.0284778.s008.docx]

#### S6 Table. Model selection results for bat species models.

| Model | AIC | ΔAIC |
| --- | --- | --- |
| carcasses ~ s(day, by = species:ecoregion) + species * ecoregion + re(site) + re(year) + offset(searches) | 38569 | 0 |
| carcasses ~ s(day, by = species) + species + s(day, by = ecoregion) + ecoregion + re(site) + re(year) + offset(searches) | 43122 | 4553 |
| carcasses ~ s(day, by = species) + species + s(day, by = ecoregion ) + ecoregion + offset(searches) | 45174 | 6605 |
| carcasses ~ s(day) + re(site) + re(year) + offset(searches) | 47227 | 8658 |
| carcasses ~ s(day) + re(site) + offset(searches) | 47399 | 8830 |
| carcasses ~ s(day, by = species) + species + offset(searches) | 47909 | 9340 |
| carcasses ~ s(day, by = ecoregion) + ecoregion + offset(searches) | 48454 | 9885 |
| carcasses ~ s(day) + re(year) + offset(searches) | 49979 | 11410 |
| carcasses ~ s(day) + offset(searches) | 50664 | 12095 |
| s=smooth term; re=random effect |  |  |
