# Supplementary material for: Seasonal patterns of bird and bat collision fatalities at wind turbines
Source: PLoS One. 2023 May 10;18(5):e0284778. doi: 10.1371/journal.pone.0284778 (PMC10171668; doi:10.1371/journal.pone.0284778)
Supplement: S7 Table — (DOCX) [file pone.0284778.s009.docx]

#### S7 Table. Model selection results for bird guild models.

| Model | AIC | ΔAIC |
| --- | --- | --- |
| carcasses ~ s(day, by = species guild:ecoregion) + species guild * ecoregion + re(site) + re(year) + offset(searches) | 11129 | 0 |
| carcasses ~ s(day, by = species guild) + species guild + s(day, by = ecoregion) + ecoregion + re(site) + re(year) + offset(searches) | 11702 | 572.7 |
| carcasses ~ s(day, by = species guild) + species guild + s(day, by = ecoregion) + ecoregion + offset(searches) | 12200 | 1070 |
| carcasses ~ s(day) + re(site) + re(year) + offset(searches) | 12359 | 1230 |
| carcasses ~ s(day) + re(site) + offset(searches) | 12366 | 1236 |
| carcasses ~ s(day, by = ecoregion) + ecoregion + offset(searches) | 12701 | 1572 |
| carcasses ~ s(day, by = species guild) + species guild + offset(searches) | 12875 | 1746 |
| carcasses ~ s(day) + re(year) + offset(searches) | 13143 | 2014 |
| carcasses ~ s(day) + offset(searches) | 13387 | 2257 |
| s=smooth term; re=random effect |  |  |
