# Supplementary material for: Seasonal patterns of bird and bat collision fatalities at wind turbines
Source: PLoS One. 2023 May 10;18(5):e0284778. doi: 10.1371/journal.pone.0284778 (PMC10171668; doi:10.1371/journal.pone.0284778)
Supplement: S8 Table — (DOCX) [file pone.0284778.s010.docx]

#### S10 Table. Significance of predictor variables for full dataset models (All) and the 50/50 split validation dataset (Split) for the all-bat/all-bird models.

| Variable | P value (All) | P value (Split) |
| --- | --- | --- |
| Day by Species group:Bats & Ecoregion:Mixed Wood Plains | < 0.001 | < 0.001 |
| Day by Species group:Birds & Ecoregion:Mixed Wood Plains | 0.0363 | 0.27 |
| Day by Species group:Bats & Ecoregion:Central Usa Plains | < 0.001 | < 0.001 |
| Day by Species group:Birds & Ecoregion:Central Usa Plains | 0.151 | 0.456 |
| Day by Species group:Bats & Ecoregion:Ozark/Ouachita-Appalachian Forests | < 0.001 | < 0.001 |
| Day by Species group:Birds & Ecoregion:Ozark/Ouachita-Appalachian Forests | < 0.001 | < 0.001 |
| Day by Species group:Bats & Ecoregion:Temperate Prairies | < 0.001 | < 0.001 |
| Day by Species group:Birds & Ecoregion:Temperate Prairies | < 0.001 | 0.0028 |
| Day by Species group:Bats & Ecoregion:West-Central Semiarid Prairies | < 0.001 | < 0.001 |
| Day by Species group:Birds & Ecoregion:West-Central Semiarid Prairies | < 0.001 | 0.0813 |
| Day by Species group:Bats & Ecoregion:South Central Semiarid Prairies | < 0.001 | < 0.001 |
| Day by Species group:Birds & Ecoregion:South Central Semiarid Prairies | < 0.001 | < 0.001 |
| Day by Species group:Bats & Ecoregion:Southern Texas Plains | < 0.001 | 0.00903 |
| Day by Species group:Birds & Ecoregion:Southern Texas Plains | 0.236 | < 0.001 |
| Day by Species group:Bats & Ecoregion:Warm Deserts | < 0.001 | < 0.001 |
| Day by Species group:Birds & Ecoregion:Warm Deserts | < 0.001 | < 0.001 |
| Random effect of site | < 0.001 | < 0.001 |
| Random effect of year | < 0.001 | < 0.001 |
