# Supplementary material for: Seasonal patterns of bird and bat collision fatalities at wind turbines
Source: PLoS One. 2023 May 10;18(5):e0284778. doi: 10.1371/journal.pone.0284778 (PMC10171668; doi:10.1371/journal.pone.0284778)
Supplement: S9 Table — (DOCX) [file pone.0284778.s011.docx]

#### S11 Table. Significance of predictor variables for full dataset models (All) and the 50/50 split validation dataset (Split) for bat species models.

| Variable | P value (All) | P value (Split) |
| --- | --- | --- |
| Day by Species:Big brown bat & Ecoregion:Central Usa Plains | < 0.001 | < 0.001 |
| Day by Species:Eastern red bat & Ecoregion:Central Usa Plains | < 0.001 | < 0.001 |
| Day by Species:Hoary bat & Ecoregion:Central Usa Plains | < 0.001 | < 0.001 |
| Day by Species:Mexican free-tailed bat & Ecoregion:Central Usa Plains | 1 | 1 |
| Day by Species:Other bats & Ecoregion:Central Usa Plains | 0.0114 | 0.124 |
| Day by Species:Silver-haired bat & Ecoregion:Central Usa Plains | < 0.001 | < 0.001 |
| Day by Species:Big brown bat & Ecoregion:Mixed Wood Plains | < 0.001 | 0.166 |
| Day by Species:Eastern red bat & Ecoregion:Mixed Wood Plains | < 0.001 | < 0.001 |
| Day by Species:Hoary bat & Ecoregion:Mixed Wood Plains | < 0.001 | < 0.001 |
| Day by Species:Mexican free-tailed bat & Ecoregion:Mixed Wood Plains | 1 | 1 |
| Day by Species:Other bats & Ecoregion:Mixed Wood Plains | < 0.001 | 0.0422 |
| Day by Species:Silver-haired bat & Ecoregion:Mixed Wood Plains | < 0.001 | < 0.001 |
| Day by Species:Big brown bat & Ecoregion:Ozark/Ouachita-Appalachian Forests | < 0.001 | < 0.001 |
| Day by Species:Eastern red bat & Ecoregion:Ozark/Ouachita-Appalachian Forests | < 0.001 | < 0.001 |
| Day by Species:Hoary bat & Ecoregion:Ozark/Ouachita-Appalachian Forests | < 0.001 | < 0.001 |
| Day by Species:Mexican free-tailed bat & Ecoregion:Ozark/Ouachita-Appalachian Forests | 1 | 1 |
| Day by Species:Other bats & Ecoregion:Ozark/Ouachita-Appalachian Forests | < 0.001 | < 0.001 |
| Day by Species:Silver-haired bat & Ecoregion:Ozark/Ouachita-Appalachian Forests | < 0.001 | < 0.001 |
| Day by Species:Big brown bat & Ecoregion:South Central Semiarid Prairies | 0.0341 | 0.45 |
| Day by Species:Eastern red bat & Ecoregion:South Central Semiarid Prairies | < 0.001 | < 0.001 |
| Day by Species:Hoary bat & Ecoregion:South Central Semiarid Prairies | < 0.001 | < 0.001 |
| Day by Species:Mexican free-tailed bat & Ecoregion:South Central Semiarid Prairies | < 0.001 | < 0.001 |
| Day by Species:Other bats & Ecoregion:South Central Semiarid Prairies | < 0.001 | < 0.001 |
| Day by Species:Silver-haired bat & Ecoregion:South Central Semiarid Prairies | 0.826 | 0.279 |
| Day by Species:Big brown bat & Ecoregion:Southern Texas Plains | 0.411 | 0.416 |
| Day by Species:Eastern red bat & Ecoregion:Southern Texas Plains | 1 | 1 |
| Day by Species:Hoary bat & Ecoregion:Southern Texas Plains | 0.00126 | 0.184 |
| Day by Species:Mexican free-tailed bat & Ecoregion:Southern Texas Plains | < 0.001 | < 0.001 |
| Day by Species:Other bats & Ecoregion:Southern Texas Plains | < 0.001 | < 0.001 |
| Day by Species:Silver-haired bat & Ecoregion:Southern Texas Plains | 1 | 1 |
| Day by Species:Big brown bat & Ecoregion:Temperate Prairies | < 0.001 | < 0.001 |
| Day by Species:Eastern red bat & Ecoregion:Temperate Prairies | < 0.001 | < 0.001 |
| Day by Species:Hoary bat & Ecoregion:Temperate Prairies | < 0.001 | < 0.001 |
| Day by Species:Mexican free-tailed bat & Ecoregion:Temperate Prairies | 1 | 1 |
| Day by Species:Other bats & Ecoregion:Temperate Prairies | < 0.001 | < 0.001 |
| Day by Species:Silver-haired bat & Ecoregion:Temperate Prairies | < 0.001 | < 0.001 |
| Day by Species:Big brown bat & Ecoregion:West-Central Semiarid Prairies | 0.958 | 0.387 |
| Day by Species:Eastern red bat & Ecoregion:West-Central Semiarid Prairies | 0.022 | 0.0683 |
| Day by Species:Hoary bat & Ecoregion:West-Central Semiarid Prairies | < 0.001 | < 0.001 |
| Day by Species:Mexican free-tailed bat & Ecoregion:West-Central Semiarid Prairies | 1 | 1 |
| Day by Species:Other bats & Ecoregion:West-Central Semiarid Prairies | 1 | 1 |
| Day by Species:Silver-haired bat & Ecoregion:West-Central Semiarid Prairies | < 0.001 | < 0.001 |
| Random effect of site | < 0.001 | < 0.001 |
| Random effect of year | < 0.001 | < 0.001 |
