# Supplementary material for: Seasonal patterns of bird and bat collision fatalities at wind turbines
Source: PLoS One. 2023 May 10;18(5):e0284778. doi: 10.1371/journal.pone.0284778 (PMC10171668; doi:10.1371/journal.pone.0284778)
Supplement: S10 Table — (DOCX) [file pone.0284778.s012.docx]

#### S12 Table. Significance of predictor variables for full dataset models (All) and the 50/50 split validation dataset (Split) for bird guild models.

| Variable | P value (All) | P value (Split) |
| --- | --- | --- |
| Day by Species guild:Grassland & Ecoregion:Mixed Wood Plains | 0.0687 | 0.504 |
| Day by Species guild:Soaring & Ecoregion:Mixed Wood Plains | 0.173 | 0.513 |
| Day by Species guild:Woodland & Ecoregion:Mixed Wood Plains | < 0.001 | 0.897 |
| Day by Species guild:Grassland & Ecoregion:Central Usa Plains | 0.229 | 0.416 |
| Day by Species guild:Soaring & Ecoregion:Central Usa Plains | 0.156 | 0.236 |
| Day by Species guild:Woodland & Ecoregion:Central Usa Plains | 0.0024 | < 0.001 |
| Day by Species guild:Grassland & Ecoregion:Ozark/Ouachita-Appalachian Forests | 0.0919 | 0.148 |
| Day by Species guild:Soaring & Ecoregion:Ozark/Ouachita-Appalachian Forests | 0.444 | 0.609 |
| Day by Species guild:Woodland & Ecoregion:Ozark/Ouachita-Appalachian Forests | < 0.001 | < 0.001 |
| Day by Species guild:Grassland & Ecoregion:Temperate Prairies | < 0.001 | 0.0385 |
| Day by Species guild:Soaring & Ecoregion:Temperate Prairies | 0.277 | 0.654 |
| Day by Species guild:Woodland & Ecoregion:Temperate Prairies | < 0.001 | 0.461 |
| Day by Species guild:Grassland & Ecoregion:West-Central Semiarid Prairies | 0.00184 | 0.00107 |
| Day by Species guild:Soaring & Ecoregion:West-Central Semiarid Prairies | 0.699 | 0.0263 |
| Day by Species guild:Woodland & Ecoregion:West-Central Semiarid Prairies | 0.0785 | 0.28 |
| Day by Species guild:Grassland & Ecoregion:South Central Semiarid Prairies | 0.00233 | 0.0017 |
| Day by Species guild:Soaring & Ecoregion:South Central Semiarid Prairies | 0.211 | 0.669 |
| Day by Species guild:Woodland & Ecoregion:South Central Semiarid Prairies | 0.00443 | < 0.001 |
| Day by Species guild:Grassland & Ecoregion:Southern Texas Plains | < 0.001 | 0.289 |
| Day by Species guild:Soaring & Ecoregion:Southern Texas Plains | 0.00516 | 0.82 |
| Day by Species guild:Woodland & Ecoregion:Southern Texas Plains | < 0.001 | 0.00538 |
| Day by Species guild:Grassland & Ecoregion:Warm Deserts | 0.319 | 0.0175 |
| Day by Species guild:Soaring & Ecoregion:Warm Deserts | 0.22 | 0.934 |
| Day by Species guild:Woodland & Ecoregion:Warm Deserts | < 0.001 | 0.112 |
| Random effect of site | < 0.001 | < 0.001 |
| Random effect of year | 0.0101 | 0.443 |
