# Supplementary material for: Seasonal patterns of bird and bat collision fatalities at wind turbines
Source: PLoS One. 2023 May 10;18(5):e0284778. doi: 10.1371/journal.pone.0284778 (PMC10171668; doi:10.1371/journal.pone.0284778)
Supplement: Attachment — Submitted filename: PONE-D-22-30951_review1.pdf [file pone.0284778.s013.pdf]

General comments

The manuscript looks at a large dataset from bird and bat collisions with wind turbines across the United States. This study provides an interesting look at the seasonal differences in fatalities of bat species and bird guilds. Overall, I have very few concerns with the manuscript. Most of my comments below are grammatical errors, or areas where a bit more information or clarification could be added. One small concern that I have is that the last sentence of the Abstract suggests that this study "highlights the value of pooling data…" but the Discussion focuses heavily on the inadequacies of the study. The authors might consider finishing the manuscript off by suggesting why they think this study is still worth being out in the scientific literature (I think it is!), rather than on why future studies might want to avoid this strategy. I don't want to suggest that the authors oversell this work, but the Discussion seems to undersell the importance of the study.

Specific comments

Lines 38 to 39 – How did the authors determine which birds belonged to which guilds? The authors might include adding reference(s).

Line 56 – Why did the spring peak exceed the fall in magnitude? Could the authors add a few more words to explain?

Line 60 – Similar comment to previous. Why do bats not show a spring peak in fatalities?

Line 182 – Remove "models" after "GAMM" to read "We used GAMMs first to describe…"

Lines 292 to 293 – "Most of the improvement in model performance seems to be related to profound differences in seasonal timing of fatalities among guilds." Perhaps I'm misunderstanding but this sentence reads awkwardly to me. Maybe change to "Most of the variation in model performance…" rather than "improvement" or change the second half of the sentence. It's the "improvement related to differences" that doesn't make sense to me.

Lines 334 to 335 – "Species that occurred across multiple ecoregions tended to show earlier fatality peaks in more northerly ecoregions." – Why? Could the authors add a few words or a sentence explaining this?

Line 357 – Why was year a factor that complicated results into practice? Explain.

Lines 359 to 360 – "Absent robust, site-specific data, longer curtailment periods will be required to account for uncertainties in when fatalities will peak at any given site in any given year." This reads awkwardly. Maybe change to "In the absence of robust, site-specific data…"?

Line 370 to 371 – "Going forward, all new studies contributed to AWWIC will include search schedules and exact dates of carcass discovery…" Why is this? Is this a new requirement of AWWIC? Might be worth stating this.

Line 398 – Remove one of these words "including specifying".

Lines 418 to 420 – "That climate change is inducing directional changes in the timing of key events in the life cycle of many organisms, including potentially in the timing of bird and bat collisions with turbines, is more difficult to address." Awkward wording. Maybe switch this around so it reads "It is more difficult to address if climate change is…" This point also seems a bit out of place. A reference to climate change seems important but a bit out of nowhere. I suggest expanding or removing.

Figure 1 – Maybe it's the quality of the figure in this version but some of the text is a bit difficult to read. Particularly the 9.2 label over the dark blue region. Perhaps this one could be switched to white text?

Figure 4 – Again, this is likely a function of low-quality images, but the colours and the boundaries of the shaded areas are difficult to see. Ignore this comment if the version that will be published is clear.
