# Supplementary material for: Seasonal patterns of bird and bat collision fatalities at wind turbines
Source: PLoS One. 2023 May 10;18(5):e0284778. doi: 10.1371/journal.pone.0284778 (PMC10171668; doi:10.1371/journal.pone.0284778)
Supplement: Attachment — Submitted filename: Reviewer Recommendation.docx [file pone.0284778.s014.docx]

**Reviewer Recommendations**

Please provide:

Please provide a detection map. Some graphic example to visualise the search radius and data collected in any typical PCM.

Please provide:

Bar graph of detected bat species/ bird guilds- overall detections- worse hit species for bats/ and worse hit guilds for birds.

Line 45 Citation

Bose A, Dürr T, Klenke RA, Henle K (2020) Predicting strike susceptibility and collision patterns of the common buzzard at wind turbine structures in the federal state of Brandenburg, Germany. PLoS ONE 15(1): e0227698. doi:10.1371/journal.pone.0227698

Line 129 Citation

Bose, A., Dürr, T., Klenke, R.A. *et al.* Collision sensitive niche profile of the worst affected bird-groups at wind turbine structures in the Federal State of Brandenburg, Germany. *Sci Rep* **8**, 3777 (2018). https://doi.org/10.1038/s41598-018-22178-z

Line 206 Citation

Bose, A., Dürr, T., Klenke, R.A. *et al.* Collision sensitive niche profile of the worst affected bird-groups at wind turbine structures in the Federal State of Brandenburg, Germany. *Sci Rep* **8**, 3777 (2018). https://doi.org/10.1038/s41598-018-22178-z

Erickson, W., Wolfe, M., Bay, K., Johnson, D. & Gehring, J. L. A comprehensive analysis of small-passerine fatalities from collision with turbines at wind energy facilities. *PLOS ONE.* **9**(9), e107491 (2014)

Line 309

Kindly change the line to “Based on data collected” and no means of verification of the statement is provided.
